# BBmix: a Bayesian beta-binomial mixture model for accurate genotyping from RNA-sequencing

**DOI:** 10.1093/bioinformatics/btad393

**Published:** 2023-06-20

**Authors:** Elena Vigorito, Anne Barton, Costantino Pitzalis, Myles J Lewis, Chris Wallace

**Affiliations:** MRC Biostatistics Unit, University of Cambridge, Cambridge CB2 0SR, United Kingdom; Division of Musculoskeletal and Dermatological Sciences, University of Manchester, Manchester M13 9PL, United Kingdom; Centre for Experimental Medicine and Rheumatology, William Harvey Research Institute, Barts and The London School of Medicine and Dentistry, Queen Mary University of London, London EC1M 6BQ, United Kingdom; Centre for Experimental Medicine and Rheumatology, William Harvey Research Institute, Barts and The London School of Medicine and Dentistry, Queen Mary University of London, London EC1M 6BQ, United Kingdom; MRC Biostatistics Unit, University of Cambridge, Cambridge CB2 0SR, United Kingdom; Cambridge Institute of Therapeutic Immunology & Infectious Disease (CITIID), Jeffrey Cheah Biomedical Centre, Cambridge Biomedical Campus, University of Cambridge, Cambridge CB2 0AW, United Kingdom

## Abstract

**Motivation:**

While many pipelines have been developed for calling genotypes using RNA-sequencing (RNA-Seq) data, they all have adapted DNA genotype callers that do not model biases specific to RNA-Seq such as allele-specific expression (ASE).

**Results:**

Here, we present Bayesian beta-binomial mixture model (BBmix), a Bayesian beta-binomial mixture model that first learns the expected distribution of read counts for each genotype, and then deploys those learned parameters to call genotypes probabilistically. We benchmarked our model on a wide variety of datasets and showed that our method generally performed better than competitors, mainly due to an increase of up to 1.4% in the accuracy of heterozygous calls, which may have a big impact in reducing false positive rate in applications sensitive to genotyping error such as ASE. Moreover, BBmix can be easily incorporated into standard pipelines for calling genotypes. We further show that parameters are generally transferable within datasets, such that a single learning run of less than 1 h is sufficient to call genotypes in a large number of samples.

**Availability and implementation:**

We implemented BBmix as an R package that is available for free under a GPL-2 licence at https://gitlab.com/evigorito/bbmix and https://cran.r-project.org/package=bbmix with accompanying pipeline at https://gitlab.com/evigorito/bbmix_pipeline.

## 1 Introduction

RNA-sequencing (RNA-Seq) is an established and popular technique with applications in transcript quantification and detection of alternative splicing, among others. More recently, a growing number of studies have developed and tested pipelines to use RNA-seq for variant and genotype calling (Quinn *et al.* 2013, Rogier *et al.* 2018, Adetunji *et al.* 2019, Brouard *et al.* 2019), including somatic variant detection and identification of cancer drivers (Akutagawa *et al.* 2022). Applications include calling variants when DNA genotypes are unavailable (Wang *et al.* 2021), studying cis-regulated genes by analysing allele-specific expression (ASE) or conducting genome-wide association studies (GWAS) in non-model species, since whole-genome sequencing is expensive and exome sequencing tools may be unavailable (Jehl *et al.* 2021). Genotype calling using RNA-seq is often more challenging than DNA sequencing: coverage can be highly variable between genes, many reads map across splice junctions making correct alignment more difficult, and cis-acting eQTLs may create unequal expression between chromosomes. Despite this, for many applications, accuracy is paramount, e.g. in ASE, miscalling a homozygous genotype as heterozygous will create a false signal of allelic imbalance.

RNA-seq genotyping pipelines employ RNA-seq-based aligners and quality control steps for filtering out potentially erroneous calls, but use the same statistical models as for DNA sequencing. While adopting these methods provides a convenient way to call variants from RNA-seq, there are sources of variation in RNA-seq data that are not accounted for. In DNA sequencing, in an ideal world reads would follow a binomial model, then, out of *n* reads covering a single nucleotide polymorhism (SNP), we would expect 0, *n*/2, and *n* to contain the alternative allele for genotypes 0, 1, and 2, respectively. Sampling variation and reference mapping bias may mean the observed heterozygote genotype count deviates from *n*/2 particularly when *n* is small, and the homozygote observations may deviate minorly from 0 and *n* due to sequencing or mapping errors. However, when *n* is large, the genotypes are expected to be clearly distinguishable. In RNA-seq, the heterozygote observation may show much greater variance, because heterozygosity at local regulatory sequences may cause overamplification of the allele on one chromosome over the allele on its homologue, with this effect varying between samples as genotypes at the regulatory sequences vary. This may push the observed heterozygote count nearer to 0 or *n*, making a miscall as a homozygote more likely, particularly when *n* is small.

In this work, we wanted to test whether directly modelling this additional variation in RNA-seq data could improve the quality of the genotype calls. To this end, we developed Bayesian beta-binomial mixture model (BBmix), a two-step method based on first modelling the genotype-specific read counts using beta-binomial distributions and then using these to infer genotype posterior probabilities. We benchmarked our method using high confidence calls from the Genome in a Bottle consortium (https://www.nist.gov/programs-projects/genome-bottle), the subset of GBR samples from the Genetic European Variation in Disease (GEUVADIS) project (McVean *et al.* 2012), for which RNA-seq data and high-quality genotype calls are publicly available and samples from the Pathobiology of Early Arthritis Cohort (PEAC) cohort (Lewis *et al.* 2019) for a ‘real data’ case study. This is a cohort of 82 adult treatment-naive rheumatoid arthritis patients for whom RNA-seq from synovial tissue samples and DNA-microarray-based genotypes are available. We found that our method generally performed better than competitors and can be easily incorporated into standard pipelines for calling variants.

## 2 Materials and methods

### 2.1 The BBmix statistical model

We model the number of reads of the alternative allele at each SNP as a mixture of three beta-binomial distributions corresponding to the three possible genotypes, conditional on the total number of reads overlapping the SNP. To allow for sequencing or alignment errors as well as reference mapping bias, the mean and variance parameters of each distribution are learned from the data. As the parameters of these distributions are shared between SNPs, we learn them from many SNPs in a Bayesian model. To maximize computational efficiency, we train the model on a random subset of SNPs and use the learnt parameters to call genotype probabilities across all SNPs.

#### 2.1.1 Fitting a beta-binomial mixture model to RNA-seq data

We selected RNA-seq reads overlapping a predefined set of feature SNPs (fSNPs) that we aim to genotype. We treat genotype as a latent variable, *G*, which corresponds to the number of alternative alleles at a given fSNP, *g* = (0, 1, 2), and we assume that the distribution of reads differs according to its value. Given fSNPs indexed by *l*, we denote *R_al_* as the number of reads overlapping its alternative allele and *R_tl_* the total number of reads mapping *s_l_*. We then fit a mixture of three beta-binomial distributions to the allelic counts of a random sample of *L* fSNPs to estimate the parameters (*μ*, vector of the mean of each component, and μ, vector of the overdispersion of each component) for the underlying distribution of genotypes. With λ a vector of the proportion of each component, we express the likelihood as where BB() is the beta-binomial distribution: which we reparameterize to:


$$L=\Pi_{l=1}^{l=L}\sum_{g=0}^{g=2}\theta_{g}BB\left(R_{al}|R_{tl},\mu_{g},\lambda_{g}\right),$$

$$f(R_{al}|R_{tl},\alpha_{g},{\beta_{g}}_{})=\frac{\left({}_{R_{al}}^{R_{tl}}\right)B(R_{al}+\alpha_{g},\;R_{tl}-R_{al}+\beta_{g})}{B(\alpha_{g},\beta_{g})}$$

$$\mu_{g}=\frac{\alpha_{g}}{\alpha_{g}+\beta_{g}},\;\lambda_{g}=\frac{1}{1+\alpha_{g}+\beta_{g}}.$$

We put the following priors:


θ∼Dirichlet(1,1,1)

α∼(1,10,499)

β∼(499,10,1)

$$\mu_{g}∼\mathrm{Beta}(\alpha_{g},\beta_{g})$$

$$\lambda_{g}∼\Gamma \left(\alpha_{g}+\beta_{g},1\right).$$

We selected the Dirichlet prior as the natural choice for a set of probabilities that sum to 1, and the informative priors for α and β were selected by visual inspection of the fit of beta distributions to the empirical distribution of reads for a collection of fSNPs with effect allele frequency (EAF) higher than 0.01 (Supplementary Fig. S1).

**Figure 1.**
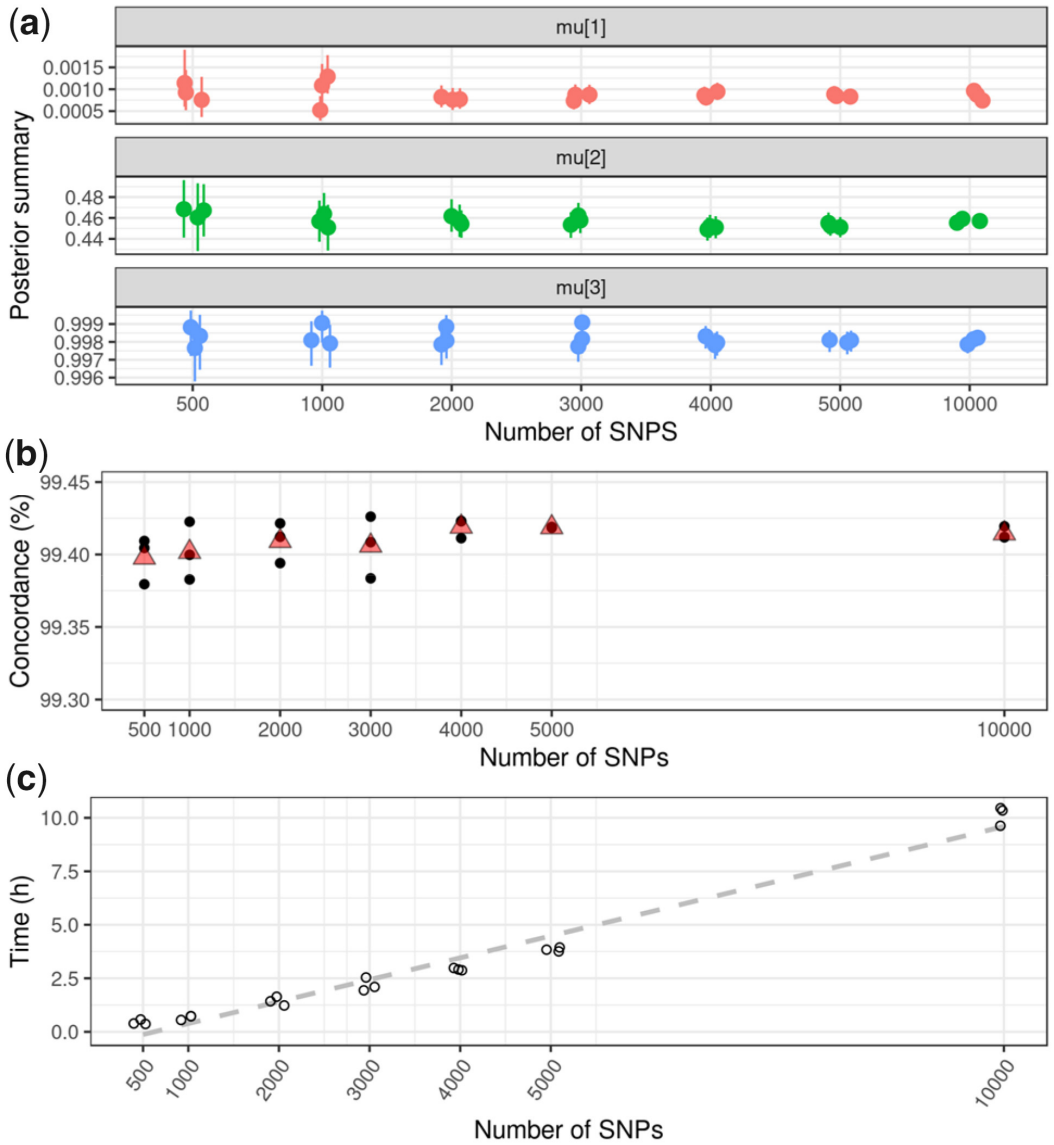
Training the beta-binomial mixture (BBmix) model with different numbers of SNPs. A random sample of RNA-seq reads for the indicated number of SNPs with at least 10 supportive reads was used to train the BBmix model. The procedure was repeated three times. (a) Mean and 95% credible interval for the posterior distribution for each of the three components of the mean parameter (mu[1–3]). (b) Concordance of calls (*y*-axis) based on the expected genotypes (dosages) called with the parameters learnt from the BBmix model trained with the indicated number of SNPs (*x*-axis). The black points correspond to each replicate and the red triangle corresponds to the mean. (c) Running time (h) for fitting the BBmix model for the indicated number of SNPs. The average time when training with 500 SNPs was 25 min; for 1000 SNPs was 46 min; for 2000 83 min; for 3000 128 min; for 4000 2.9 h; for 5000 SNPs 4 h, and for 10 000 SNPs 10 h. The dashed line corresponds to the linear regression line.

We estimated μ and λ by Markov chain Monte Carlo implemented in Stan (Stan Development Team, 2018). We checked convergence using Stan built-in *n_eff* and *Rhat* statistics as well as visual examination. We obtained 4000 posterior observations sampled from four chains for each parameter. For each chain, we discarded the first 1000 observations.

#### 2.1.2 Calling genotypes

We called genotypes on a per-individual basis. Details can be found in Supplementary Note S1.

### 2.2 Datasets

We used RNA-seq data from lymphoblastic cells lines derived from NA12878 and 13 additional samples (study E-MTAB-1883) with the high confidence genotype calls from the Genome in a Bottle consortium for sample NA12878 used as a gold standard. Note that only non-reference homozygote genotypes are available in the vcf files for this data source. The Genome in a Bottle consortium also provides BED files with high confidence sequencing data for defined regions across the genome for sample NA12978. Although it would be safe to assume that any site included within these high-confidence regions for which variants have not been called are homozygous reference i we did not include those positions in our analysis. We also selected a subsample of 86 individuals of European ancestry coded as GBR from the GEUVADIS for which genotypes and RNA-seq data are publicly available and genotypes and RNA-seq data from the PEAC study. Details for data sources both for genotypes and RNA-seq can be found in Supplementary Note S2.

### 2.3 RNA-seq data processing

We applied the GATK pipeline for RNA-seq short variant discovery (SNPs + Indels, https://gatk.broadinstitute.org/hc/en-us/articles/360035531192-RNAseq-short-variant-discovery-SNPs-Indels-) for read alignment and data cleanup (removal of duplicates and base quality recalibration**)** before calling variants. Details on the implementation are described in Supplementary Note S3.

### 2.4 Comparison to alternative methods

We validated BBmix against three other popular methods: HaplotypeCaller (Brouard *et al.* 2019), Mpileup from Bcftools (Li 2011), and FreeBayes (Garrison and Marth 2012) which have been applied for RNA-seq genotyping (Quinn *et al.* 2013, Rogier *et al.* 2018, Adetunji *et al.* 2019, Wang *et al.* 2021). We also compared BBmix with a naive method based on applying thresholds on the proportion of reads mapping the alternative allele; we referred to this method as ‘Count threshold’. This comparison allowed us to directly assess the benefit of implementing a statistical model for modelling read counts. The alignment output from the GATK pipeline was used as input for calling genotypes with BBmix, Count threshold, Haplotypecaller, and Mpileup/bcftools. For FreeBayes, the base quality recalibration step was excluded as it is not recommended (https://github.com/freebayes/freebayes). Each method was run with default parameters. Details of the implementation are in Supplementary Note S4. We compared hard calls for exonic variants annotated in the 1000 Genome project with allele frequency higher than 0.01 and excluded regions of the genome with known mapping bias against the corresponding gold standard (Supplementary Note S4). Thus, for each method we have quantified the number of concordant, discordant, or missing calls, and reported the accuracy and total number of calls. For the PEAC gold standard for which probabilistic genotype calls were available, we selected the subset for which genotypes were called with probability 1.

### 2.5 Code availability

The R package with the code for genotyping using bbmix can be found at https://gitlab.com/evigorito/bbmix and https://cran.r-project.org/package=bbmix. The pipeline for preparing the inputs for running bbmix can be found at https://gitlab.com/evigorito/bbmix_pipeline. The pipeline and code used for preparing the manuscript is at https://gitlab.com/evigorito/bbmixpaper_pipeline. The versions of all the software tools used for producing the bbmix R-package, pipeline, and manuscript are listed in Supplementary Note S5.

## 3 Results

### 3.1 Determining the number of SNPs for training the beta-binomial mixture model

We first evaluated the effect of the number of SNPs used to train the beta-binomial mixture (BBmix) model on the accuracy of genotype calls. To this end, three sets of 500, 1000, 2000, 3000, 4000, 5000, or 10 000 SNPs with at least 10 mapping reads (*21 sets* in total) were randomly selected from the Genome in a bottle sample NA12878. We fit the BBmix model independently on each subset. As expected, increasing the number of SNPs resulted in a narrower posterior distribution of the mean parameter of each component (Fig. 1a), while the posterior mean for heterozygous calls departed from 0.5, suggestive of over-representation of the reference allele consistent with reference mapping bias (Fig. 1a, medium panel). Note that each component of the model corresponds to each possible genotype. Increasing the number of training SNPs also resulted in overall small gains in concordance between genotype dosages estimated by BBmix and the high-confidence calls available from the Genome in a bottle consortium until a plateau was reached at about 4000 SNPs (Fig. 1b). For subsequent analysis, we trained our model with 1000 SNPs, which we considered a good trade-off between concordance and computational time (mean time = 46 min, Fig. 1c).

Some analyses require hard calls instead of genotype dosages. To define hard calls, we assigned the most likely genotype when its probability is above a predefined threshold. We tested thresholds of 0.90, 0.95, and 0.99 for sample NA12878 and chose to favour accuracy over number of calls. We selected 0.99 as at depth 10 the concordance was 99.82% (0.1% increase relative to 0.9) while the loss in the number of calls was 0.15% (9303 total calls, Supplementary Fig. S2). In subsequent analyses, when applying the BBmix model on hard calls we selected 0.99 as the probability threshold.

**Figure 2.**
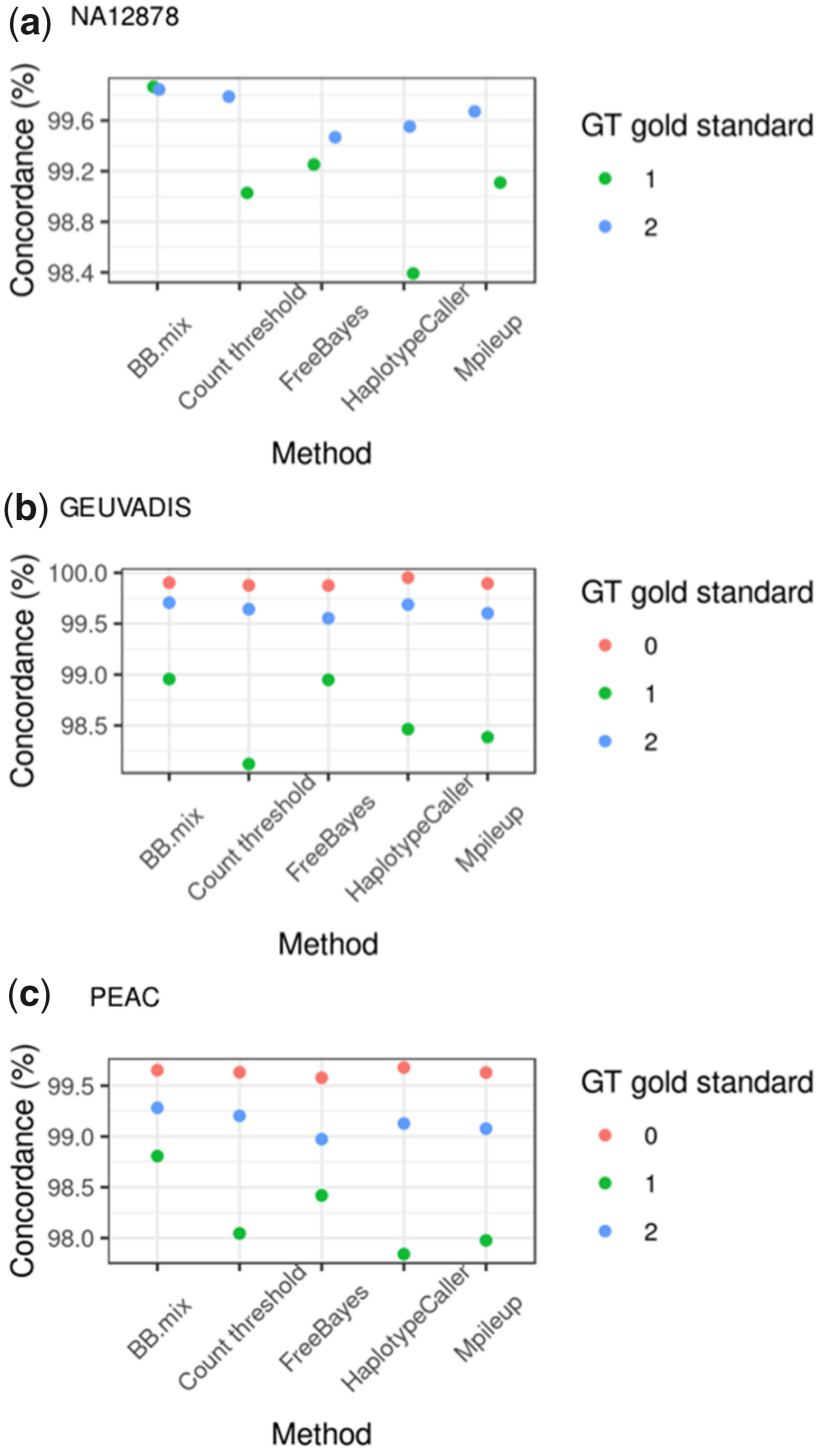
Concordance of calls by genotype. For each method, the number of concordant calls relative to the gold standard for those SNPs with at least 10 supporting reads was calculated. The analysis was done for (a) Genome in a Bottle sample NA12878, (b) GEUVADIS samples, and (c) PEAC samples. Note that for sample NA12878, the gold standard has only calls for heterozygous (1) and homozygous alternative (2) calls.

### 3.2 Benchmarking the BBmix model

We applied HaplotypeCaller, Mpileup from Bcftools, FreeBayes, Count threshold, and BBmix to three datasets: the highly curated genome in a bottle sample, a subset of the GEUVADIS samples with high-quality genotypes and samples from the PEAC study which were genotyped by DNA-microarrays.

**Figure 3.**
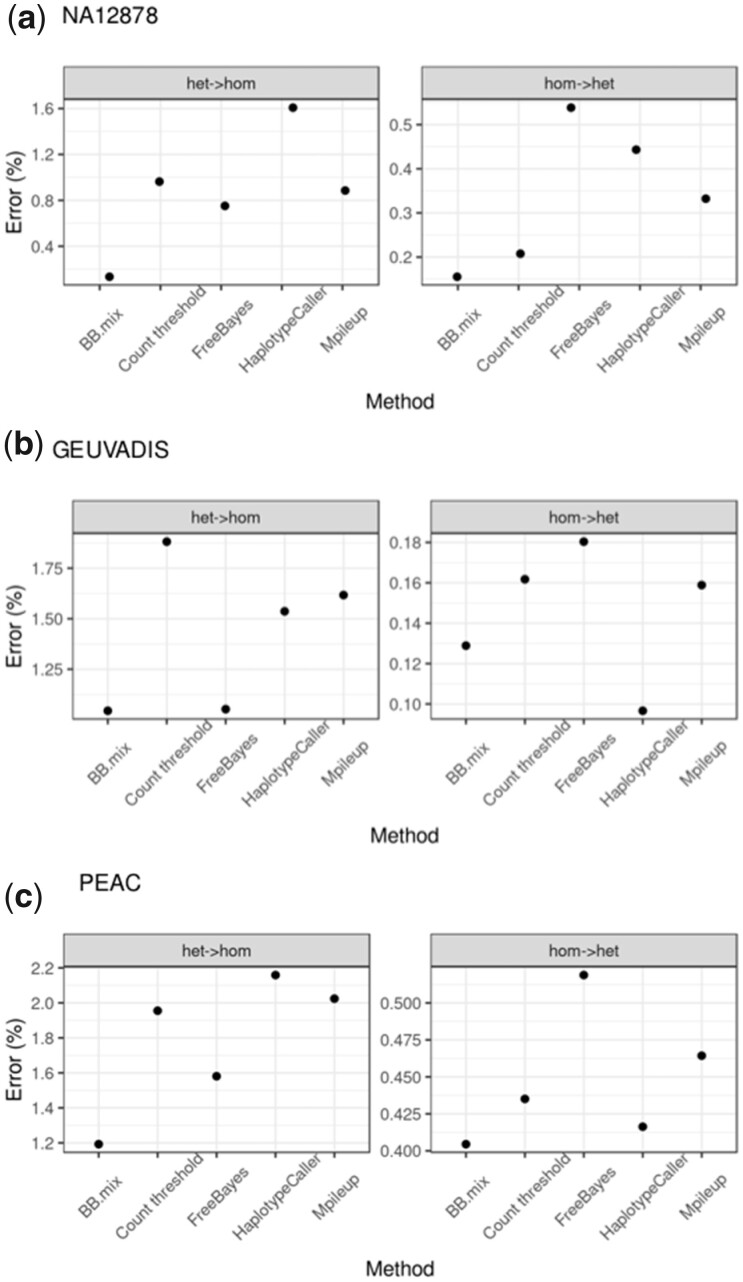
Genotype error type by method. For each method, it is shown the percentage or erroneous calls for the indicated type (heterozygous in gold standard but homozygous by method [het -> hom] or vice versa). Only SNPs with at least 10 supporting reads were considered. (a) Genome in a Bottle sample NA12878, (b) GEUVADIS samples, and (c) PEAC samples. Note that for sample NA12878 the gold standard has only calls for heterozygous (1) and homozygous alternative (2) calls.

We first assessed the accuracy of the calls stratified by genotype across the different datasets. For this analysis, we hard called genotypes for SNPs with at least 10 supporting reads, which we considered as a good trade-off between coverage and accuracy (Supplementary Figs S3–S5). Homozygous reference calls tend to be the most accurate (>99.6%) with the lowest variance across methods (Fig. 2), while heterozygous calls are generally more prone to error (accuracy < 99.0% in GEUVADIS and PEAC) and showed the highest variability across methods (Fig. 2). Overall, BBmix was generally superior to competitor methods, with the main gains in the accuracy of heterozygous calls (Fig. 2). Next, we assessed the prevalence of homozygous calls in the gold standard called heterozygous by methods (homozygous to heterozygous errors) and vice versa (heterozygous to homozygous errors). Overall, we observed a better performance of BBmix in both types of errors with the exception of GEUVADIS on homozygous to heterozygous errors with HaplotypeCaller performing better (Fig. 3). FreeBayes was consistently worst for homozygous to heterozygous errors while HaplotypeCaller had the worst heterozygous to homozygous error rate for both NA12878 and PEAC (Fig. 3). Last, we compared methods performance across a wide range of read depth threshold on aggregated genotype calls. The accuracy of BBmix genotype calls was generally superior to competitor methods though the effect appeared modest especially for GEUVADIS and PEAC (Supplementary Figs S3–S5). This is because roughly 60% of the calls were homozygous reference (Supplementary Tables S1–S3) which showed the most accuracy and least variability across methods, while for NA12878 only heterozygous or homozygous-alternative calls were compared. As expected, increasing the depth threshold decreased the gap in accuracy between BBmix and Count threshold. We also compared BBmix to the Count threshold method using additional thresholds (0.2, 0.15, 0.05, and 0.001) in addition to our default of 0.1. The highest accuracy was observed with thresholds 0.1 and 0.05 across all datasets (Supplementary Figs S6–S8). Last, for sensitivity analysis for BBmix hard calls, we compared the accuracy of BBmix using thresholds of 0.9 and 0.95, in addition to our default of 0.99 (selected based on sample NA12878 in Supplementary Fig. S2). As with sample NA12878, changing the threshold even to 0.9 produced a small drop in accuracy, which was more noticeable at lower depth (from 99.7% to 99.6% in GEUVADIS and from 99.30% to 99.28% in PEAC, Supplementary Figs S9 and S10, respectively).

With regards to the number of calls made by each method, HaplotypeCaller had the highest number of calls, about 20%–30% higher than BBmix, with the main gain being on homozygous reference calls (Supplementary Tables S1–S3 and Supplementary Figs S3b–S5b). For the subset of calls that were validated in the gold standard, Mpileup had the highest number, between 10% and 25% higher than BBmix (Supplementary Figs S3b–S5b). Overall, BBmix accuracy was generally superior at the cost of lower number of calls.

### 3.3 Exchangeability of model parameters between samples

**Figure 4.**
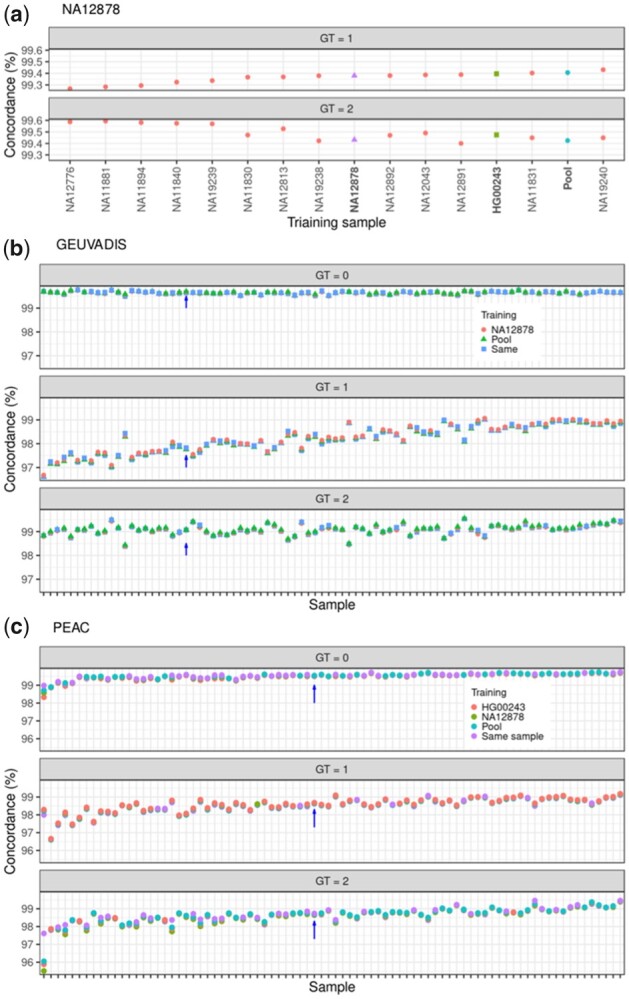
Exchangeability of model parameters. (a) The BBmix model was trained with each of the E-MTAB-1883 samples, a pool of those samples or a randomly selected external GEUVADIS sample (indicated in the *x*-axis). Genotypes were called in sample NA12878 and the concordance against the gold standard stratified by genotype is shown in the *y*-axis. (b) The BBmix model was trained with each of the GEUVADIS samples, a pool of them or the external sample NA12878. For each sample (*x*-axis), the plot shows the concordance on genotypes (*y*-axis) called with the same sample used for training the pool or NA12878. The arrow indicates sample HG00243 to ease comparison with Supplementary Fig. S6. (c) Same as in (b) except that genotypes for the PEAC samples were additionally called using HG00243 as an external sample. The arrows indicate sample QMUL2009047 to ease comparison with Supplementary Fig. S6.

Thus far we trained the BBmix model repeatedly for each sample under consideration. To save computational time, we could train the model with a sample of reads from the pooled samples to learn the model parameters μ and λ and then call genotypes in each sample. Even better would be to have default parameters from an external sample and completely avoid model training. We assessed those options as follows: for sample NA12878, we trained the model with each of the 13 samples that were RNA-sequenced together with NA12878, with a pool of reads from all of the samples or using a randomly selected GEUVADIS external sample. We then called genotypes in NA12878 using the aforementioned scheme for model training and assessed genotype accuracy against the gold standard. The accuracy on genotypes was very robust to the source of reads for parameter training, ranging from 99.25% to 99.60% for heterozygous calls and 99.40% to 99.60% for homozygous alternative calls, and very close to the model trained with NA12878 reads, 99.38% for heterozygous and 99.44% for homozygous alternative calls (Fig. 4a). Next, we extended the analysis to the GEUVADIS subcohort by training the model with a pool of reads. We then called genotypes for each sample using the model trained either with the pool of reads, the external sample NA12878 or with the same sample used to fit the model. Again, the concordance of calls across all genotypes was very similar regardless of the source of reads used for training (Fig. 4b). Last, the same analysis performed in PEAC produced the same pattern, with very high concordance on genotype calls even when training with external samples (Fig. 4c).

Prompted by the high level of genotype concordance regardless of training sample, we compared the distribution of the posterior samples for the model parameters trained with either a pool of the respective study samples with NA12878, a representative sample for GEUVADIS or PEAC. In all examples, we observed a mixed pattern with some parameters showing similar distributions but clear departures from the identity line as well (Supplementary Fig. S11).

Overall, genotype calls appeared to be robust with regards to the source of reads used for model training.

## 4 Discussion

Current pipelines for calling variants from RNA-seq rely on data cleaning and algorithms designed for DNA sequencing, ignoring ASE and reference mapping bias. In this study, we provide proof of concept that calling genotypes from RNA-seq can be improved by modelling intrinsic sources of variation in RNA-seq read counts.

Our motivation to call variants from RNA-seq was to detect expression quantitative trait loci (eQTLs) using ASE, which requires genotyping of exonic SNPs (Castel *et al.* 2015). This is because calling variants from RNA-seq has the potential to increase coverage for variants overlapping expressed genes. Moreover, genotyping errors arising from DNA-based genotyping may introduce bias. For example, if homozygous exonic SNPs are mistyped as heterozygous, the RNA-seq reads will appear to show a strong allelic imbalance. We showed here that our method generally reduced this type of error compared with the other methods and performed particularly well on heterozygous calls, which is likely due to our modelling strategy accounting for noise in RNA-seq reads.

Our contribution shows that accounting for bias in RNA-seq can improve genotyping accuracy and we provide a statistical framework for modelling such data, in the case of the most carefully curated gold standard, the Genome in a Bottle sample, we reduced the proportion of erroneous calls over 4-fold compared with the next best method. Even if BBmix tends to increase accuracy, this occurs at the cost of reduced genotype calls made. Thus, the method is designed to prioritize accuracy over volume. While our method was not designed as a standalone pipeline for discovery, our modelling approach has potential to be integrated within current algorithms such as HaplotypeCaller, Mpileup, or FreeBayes to tailor those methods for RNA-seq. Although Bayesian approaches tend to be computationally expensive, we showed that a modest number of training SNPs (1000) tends to be sufficient to learn the model parameters in less than an hour, and that genotyping was very consistent even when using an external sample, which completely eliminates the need for model training. Thus, our work presents a method tailored for genotyping using RNA-seq and also highlights that the current methods for variant-calling using DNA could be adapted for RNA-seq by modelling RNA-seq count biases.

## Supplementary Material

btad393_Supplementary_Data

## Data Availability

The “Genome in a bottle” consortium has developed a pipeline integrating sequencing data generated by multiple technologies to produce a list of high confident heterozygous and homozygous alternative variant calls widely used for benchmarking and validation of variant calling pipelines https://www.nist.gov/programs-projects/genome-bottle. The sample employed is NA12878/HG001 from the HapMap project https://www.genome.gov/10001688/international-hapmap-project. Genotype calls were downloaded from ftp://ftp-trace.ncbi.nlm.nih.gov/giab/ftp/release//NA12878_HG001/latest/GRCh37/HG001_GRCh37_GIAB_highconf_CG-IllFB-IllGATKHC-Ion-10X-SOLID_CHROM1-X_v.3.3.2_highconf_PGandRTGphasetransfer.vcf.gz. This corresponds to version v3.3.2. In addition, RNA-seq data produced from a lymphoblastic cell line derived from sample NA12878 in addition to 13 other samples from the 1000 Genome project were available in the study E-MTAB-1883 at array express https://www.ebi.ac.uk/arrayexpress/experiments/E-MTAB-1883/. We downloaded RNA-seq data from 86 GEUVADIS lymphoblastic cell line samples with EUR ancestry (GBR code) from ArrayExpress (E-GEUV-1). Genotypes by DNA sequencing are publicly available from the 1000 Genome consortium https://www.internationalgenome.org/data-portal/data-collection at https://www.internationalgenome.org/data-portal/data-collection/phase-3. For our “real data” example we used RNA-seq samples from synovial tissue for 82 samples of the Pathobiology of Early Arthritis Cohort (PEAC) study https://www.ncbi.nlm.nih.gov/pmc/articles/PMC6718830/#mmc1 available at array express (E-MTAB-6141) https://www.ebi.ac.uk/arrayexpress/experiments/E-MTAB-6141/samples/ DNA-microarray. Genotypes by DNA-microarray are available upon reasonable request.The file used to exclude difficult to map regions in the chromosome was downloaded from phASER https://github.com/secastel/phaser/tree/master/phaser at https://www.dropbox.com/s/fbfntaa4oc75x6m/hg19_hla.bed.gz?dl=0.

## Web resources

R https://cran.r-project.org/.

BBmix https://gitlab.com/evigorito/bbmix.

bcftools https://samtools.github.io/bcftools/bcftools.html.

GATK https://gatk.broadinstitute.org/hc/en-us.

FreeBayes https://github.com/freebayes/freebayes.

BBmix pipeline for input preparation https://gitlab.com/evigorito/bbmix_pipelineCode used for the analysis https://gitlab.com/evigorito/bbmixpaper_pipeline.
